# The decrease in zinc‐finger E‐box‐binding homeobox‐1 could accelerate steroid‐induced osteonecrosis of the femoral head by repressing type‐H vessel formation via *Wnt/β‐catenin* pathway

**DOI:** 10.1002/ame2.12507

**Published:** 2024-12-16

**Authors:** Guangyang Zhang, Yuanqing Cai, Jialin Liang, Zhaopu Jing, Wang Wei, Leifeng Lv, Xiaoqian Dang, Qichun Song

**Affiliations:** ^1^ Orthopedic Center, The Second Affiliated Hospital of Xi'an Jiaotong University Xi'an China

**Keywords:** steroid‐induced osteonecrosis of the femoral head, type‐H vessel, Wnt/β‐catenin pathway, zinc‐finger E‐box‐binding homeobox‐1

## Abstract

**Background:**

Zinc‐finger E‐box‐binding homeobox‐1 (*ZEB1*) is predominantly found in type‐H vessels. However, the roles of *ZEB1* and type‐H vessels in steroid‐induced osteonecrosis of the femoral head (SONFH) are unclear.

**Methods:**

Human femoral heads were collected to detect the expression of *ZEB1* and the levels of type‐H vessels. Then, the SONFH model was developed by injecting C57BL/6 mice with lipopolysaccharide and methylprednisolone. Micro‐computed tomography, angiography, double calcein labeling, immunofluorescence, immunohistochemistry, quantitative real‐time polymerase chain reaction, and Western blotting were performed to detect the expression of *ZEB1*, the *Wnt/β‐catenin* pathway, type‐H vessels, and the extent to which *ZEB1* mediates angiogenesis and osteogenesis. Human umbilical vein endothelial cells were also used to explore the relationship between *ZEB1* and the *Wnt/β‐catenin* pathway.

**Results:**

We found that *ZEB1* expression and the formation of type‐H vessels decreased in SONFH patients and in a mouse model. The number of vascular endothelial growth factors in the femoral heads also decreased. Moreover, the bone mineral density, trabecular number, mineral apposition rate, and expression of genes related to osteogenesis decreased. After *ZEB1* knockdown, angiogenesis and osteogenesis decreased. However, the numbers of type‐H vessels and the extent of angiogenesis and osteogenesis improved after activation of the *Wnt/β‐catenin* pathway.

**Conclusions:**

The ZEB1 expression decreased in SONFH, causing a decrease in type‐H vessel, and it mediated angiogenesis and osteogenesis by regulating the *Wnt/β‐catenin* pathway, ultimately accelerating the process of SONFH.

## Graphical abstract

The application of glucocorticoid could restrain the expression of *ZEB1*, causing a decrease in the expression of *β‐catenin*, transcription factor‐4, *C‐Myc*, and other key genes in the *Wnt/β‐catenin* pathway, ultimately damaging the formation of type‐H vessels and aggravating necrosis of the femoral head. However, activation of the *Wnt/β‐catenin* pathway promotes the formation of type‐H vessels and prevents SONFH (steroid‐induced osteonecrosis of the femoral head).

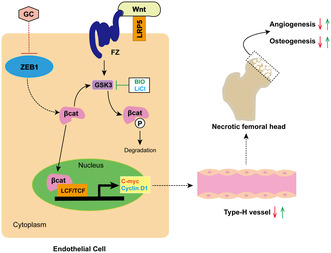

## 1 INTRODUCTION

Nontraumatic osteonecrosis of the femoral head (ONFH) is a severe condition that typically impacts individuals aged <50 years, leading to structural changes in the hip, collapse of the femoral head, and impaired joint function. A previous study revealed that more than 10 000 individuals in the United States are diagnosed with ONFH annually, accounting for nearly 10% of total hip arthroplasties.^1^ Furthermore, the number of Chinese patients affected by this disease reached 8.12 million.^2^ Steroid‐induced osteonecrosis of the femoral head (SONFH) was initially reported in 1953 and has emerged as the predominant cause of ONFH in recent years.^3^ A variety of theories, ranging from lipid metabolism disorders, a decreased osteogenic potential of bone marrow mesenchymal stem cells (BMSC), inadequate blood supply, inflammation, and cell apoptosis have been proposed for the pathogenesis of SONFH. However, the precise pathological mechanisms remain elusive. Therefore, further research on the pathogenesis of SONFH is needed to develop efficient treatments for patients with this disease.

Bone is a highly vascularized tissue that enables it to receive vital nutrients, oxygen, growth factors, and hormones from blood circulation. Recent studies have revealed that osteogenesis and angiogenesis are consistently interconnected during the process of bone modeling and remodeling, namely angiogenic–osteogenic coupling.^4^ Type‐H vessels, a distinct subtype of capillaries characterized by elevated levels of platelet endothelial cell adhesion molecule‐1 (*CD31*) and endomucin (*EMCN*), have been demonstrated to coordinate the interplay between osteogenesis and angiogenesis. A decrease in type‐H vessels was shown to be associated with many orthopedic disorders. Previous studies have shown a notable reduction in the quantity of type‐H vessels and coupled Osterix+ osteoprogenitors within the hypofunctional periodontal ligament in mice with alveolar bone disuse osteoporosis.^5^ Li et al. demonstrated that injecting slit guidance ligand 3 could improve the integration of tendon and bone, possibly by modulating the coordination between angiogenesis and osteogenesis mediated by type‐H vessels.^6^ Moreover, metformin has been shown to hold promise as a treatment option for fractures. This is because it can stimulate the formation of type‐H vessels by repressing the activity of *YAP1/TAZ*.^7^ Another study successfully constructed a polycaprolactone/hydroxyapatite‐iminodiacetic acid‐deferoxamine scaffold and revealed that it could significantly facilitate the development of type‐H vessels and the expression of coupling factors to stimulate osteogenesis, thereby increasing the regeneration of substantial bone defects in rats.^8^ However, the study investigating the role of type‐H vessels in the pathogenesis of SONFH is rare.

The *Wnt/β‐catenin* pathway plays a critical role in bone biology, including bone formation, remodeling, and homeostasis. Recently, it has been found to be intricately linked to the pathogenesis of SONFH and the development of type‐H vessels. The expressions of Runt‐related transcription factor 2 (*Runx2*) and *β‐catenin* in the necrotic region of the femoral head were considerably lower than those in the normal region. This suggested that the *Wnt/β‐catenin* pathway was compromised in the necrotic area.^9^ Furthermore, glycyrrhizic acid was shown to modify the differentiation commitment of BMSCs under hyper‐oxidative stress via activating the *Wnt/β‐catenin* pathway, ultimately preventing the development of SONFH.^10^ Another study revealed that Astragalus polysaccharide could ameliorate SONFH by regulating the miR‐200b‐3p‐mediated *Wnt/β‐catenin* signaling.^11^ The relationships between *Wnt/β‐catenin* signaling and the processes of osteogenesis and angiogenesis have also been well investigated. Shen et al. revealed that epidermal growth factor‐like domain‐containing protein 6 could stimulate angiogenesis and osteogenesis through *Wnt/β‐catenin* signaling and enhance the formation of type‐H vessels in a tibia distraction osteogenesis model.^12^ Wang et al. constructed a novel decellularized matrix and reported that it could accelerate regeneration of bone defects via osteoclastogenesis, angiogenesis, and neurogenesis by activating *Wnt* signaling.^13^

Zinc‐finger E‐box‐binding homeobox‐1 (*ZEB1*) is a zinc finger transcription factor that initiates the epithelial–mesenchymal transition process and plays a crucial role in wound healing, fibrosis, and metastasis, as well as growth, development, and various pathological conditions.^14^ Previous evidence demonstrated that *ZEB1* was mainly expressed in type‐H vessels in human and mouse bones, and the specific deletion of *ZEB1* in endothelial cells of mice decreased type‐H vessel formation in the bone, causing reduced osteogenesis.^15^ Another study revealed that a reduction in *ZEB1*‐mediated vascular endothelial growth factor (*VEGF*) transcription could impair angiogenesis.^16^ Similarly, Zhou et al. prepared a GM/Ac‐CD/rGO hydrogel and revealed that it could promote osteogenesis and angiogenesis coupling that mediated type‐H vessels via stimulating the *ZEB1/Notch1* pathway.^17^ Additionally, *ZEB1* can interact with transcription factor 4 (*TCF‐4*) to regulate the transcriptional activity of *Wnt/β‐catenin*‐related genes.^18^ *ZEB1* can activate the *Wnt/β‐catenin* signaling pathway by increasing the levels of *β‐catenin*, *C‐Myc*, and *cyclin D1*, thereby promoting cell proliferation and migration and inhibiting apoptosis.^19^ Therefore, *ZEB1* plays an important role in osteogenesis and angiogenesis; however, its specific involvement in SONFH remains uncertain and requires additional investigation.

## 2 METHODS

### 2.1 Femoral head collection

The femoral heads of humans were collected from patients who underwent total hip replacement. Femoral heads from SONFH patients classified as grade 3 or 4 based on the Ficat classification system were collected, and femoral heads from femoral neck fracture patients were selected as controls. Patients with previous bone disease or other physical disease were excluded. Moreover, those who disagreed with the research methods were also excluded. All femoral heads were stored in liquid nitrogen for subsequent analysis.

### 2.2 Animal models

Male C57BL/6 mice were obtained from the Laboratory Animal Center of Xi'an Jiaotong University and housed in specific pathogen‐free conditions (temperature: 20–25°C, humidity: 50%–60%) with four mice per cage. The mice were divided into four groups randomly after 7 days of adaptive feeding. (1) In the SONFH group, the mice were initially treated with lipopolysaccharide (20 μg/kg, Sigma‐Aldrich, MO, USA) via intraperitoneal injection daily for a duration of two consecutive days. Then, methylprednisolone (100 mg/kg, Sigma‐Aldrich) was injected into the gluteus once a day for 2 weeks and once every other day for an additional 6 weeks. In addition, the mice in this group underwent a sham operation. (2) SONFH + *ZEB1* knockdown group (SONFH+si‐*ZEB1*): the mice were subjected to the same treatments as those in the SONFH group. Moreover, rLV‐shRNA1‐m*ZEB1*‐985 (1 × 10^8^ TU/mL), prepared by Bio‐Tower Biotechnology (Wuhan, China), was injected once a week (1 × 10^6^ TU each time) into the femoral bone marrow cavity through the femoral intercondylar fossa after anesthesia by intraperitoneal injection of 50 mg/kg of sodium pentobarbital (Sigma‐Aldrich) for the last 4 weeks. (3) SONFH + *ZEB1* knockdown + lithium chloride (LiCl) group (SONFH+si‐*ZEB1*+LiCl): LiCl is an activator of the *Wnt/β‐catenin* pathway that inhibits the activity of *GSK‐3β*. The mice underwent the same procedure as the SONFH+si‐*ZEB1* group. Additionally, LiCl (200 mg/kg, Sigma‐Aldrich) was administered by gavage once a day from the seventh week to the end of the experiment. (4) Control group: the mice in the other groups were treated with an equal volume of normal saline, and a sham operation was performed. All the mice received an overdose of anesthesia by intraperitoneal injection of 100 mg/kg of sodium pentobarbital (Sigma‐Aldrich) and then killed in the eighth week, and the femoral heads were obtained and used for further analysis.

### 2.3 Cell culture

Human umbilical vein endothelial cells (HUVEC), obtained from the Shanghai National Stem Cell Translational Resource Center (Shanghai, China), were cultured at 37°C in 5% CO_2_ in an endothelial cell medium and passaged at 1:3 with 0.25% trypsin. The HUVECs were divided into four groups: (1) the control group, in which the cells were treated with equal amounts of general control lentivirus, was used as the experimental group. (2) In the *ZEB1* knockdown group (si‐*ZEB1*), the cells in each well were treated with 50 μL of lentivirus (1 × 10^8^ TU/mL, rLV‐shRNA1‐m*ZEB1*‐985, Bio‐Tower) for *ZEB1* knockdown. After 48 h of culture and cell passage, the affected HUVECs were reseeded in six‐well plates at a density of 5 × 10^5^ cells per well. Then, 6‐bromoindirubin‐3′‐oxime (BIO, 5 μM, Sigma‐Aldrich) was applied to activate the *Wnt/β‐catenin* pathway. (3) In the control + BIO group, the cells were initially treated as the control group and then treated with BIO. (4) *ZEB1* knockdown+BIO group (si‐*ZEB1*+BIO): the cells in this group were considered as the si‐*ZEB1* group; then BIO was used. After a 72‐h incubation period, all the HUVECs were collected for further analyses.

### 2.4 Quantitative real‐time polymerase chain reaction

Total RNA was extracted using TRIzol (Thermo Fisher Scientific, MA, USA) according to the manufacturer's protocol. Then, a Nanodrop (Invitrogen, USA) was used to measure the RNA concentration, and PrimeScript RT Master Mix kits (Takara, Japan) were used for complementary DNA synthesis in accordance with the standard protocol. Quantitative real‐time polymerase chain reaction (RT‐qPCR) analysis was performed on a Bio‐Rad CFX96 qPCR instrument (Bio‐Rad, Hercules, USA) with an SYBR Select Master Mix kit (Vazyme, Nanjing, China). Glyceraldehyde 3‐phosphate dehydrogenase was used as the internal control. The primer sequences for the genes investigated in this study are presented in Table S1.

### 2.5 Western blotting

The proteins were extracted using the standard procedure and quantified using the BCA Protein Assay Kit (Beyotime, Shanghai, China). The proteins were diluted to equal concentration using loading buffer, loaded onto sodium dodecyl sulfate‐polyacrylamide gel electrophoresis gel, and subsequently transferred to polyvinylidene fluoride (PVDF) membranes (Millipore, USA). After being blocked with 5% bovine serum albumin (Sigma), the proteins were incubated with primary antibodies overnight at 4°C. The PVDF membranes were then washed and incubated with secondary antibodies for 2 h at room temperature. An enhanced chemiluminescence assay (Millipore Corporation) was used for identifying the proteins. The primary antibodies employed in this study are presented in Table S2.

### 2.6 Hematoxylin and eosin, immunofluorescence, and immunohistochemistry staining

The femoral heads were dissociated and then fixed in 10% formalin for 48 h. Subsequently, they were decalcified in a water bath at 37°C with 10% ethylenediaminetetraacetic acid (EDTA). The EDTA solution was replaced every 3 days until the specimens became soft. Then the femoral heads were embedded in paraffin and sectioned (5 μm) following standard procedures; the sections were stained with hematoxylin and eosin (HE, Sigma). The bone lacunae and empty bone lacunae were quantified in three randomly selected visual fields under a 200× microscope in HE‐stained sections. The percentage of empty bone lacunae was calculated by the ratio of empty bone lacunae to total bone lacunae.

The sections for immunofluorescence (IF) staining were prepared following the same protocol as for HE staining. After the dewaxing and antigen retrieval procedures, normal goat serum was applied at room temperature for 30 min to minimize nonspecific staining. The sections were then incubated overnight at 4°C with primary antibodies against *ZEB1*, *EMCN*, *CD31* or *VEGF*, as specified by the manufacturer's instructions. Subsequently, the sections were incubated with secondary antibodies labeled with Cy3 or fluorescein isothiocyanate, and nuclear staining was performed using 4′,6‐diamidino‐2‐phenylindole. The sections were observed using a fluorescence microscope (Leica DMI6000B, Wetzlar, Germany) under dark conditions, and the sections were scanned using a slicing scanner (Pannoramic MIDI, 3DHISTECH, Budapest, Hungary).

The preparation of sections, dewaxing, and antigen retrieval for immunohistochemistry (IHC) were the same as those for IF staining. Then, the sections were treated with 3% hydrogen peroxide at room temperature for 15 min to inhibit endogenous peroxidase activity. Subsequently, the sections were incubated overnight at 4°C with the appropriate diluted primary antibodies against alkaline phosphatase (ALP), Runx2, or Osterix. After incubation, the sections were washed and then incubated again with the corresponding secondary antibodies at 37°C for 30 min. Then, the sections were stained with diaminobenzidine and counterstained with hematoxylin. Finally, the dried sections were viewed under a microscope, and images were obtained. The primary antibodies used are presented in Table S2.

### 2.7 Micro‐computed tomography and micro‐computed tomography–based angiography

The mouse femoral heads were first removed, fixed in 10% formalin for 48 h, soaked in 75% alcohol overnight, and scanned using micro‐computed tomography (CT) at 70 kV in a high‐resolution system to obtain the original images. The standard body model was also scanned under the same conditions for calibration. Next, the original images were reconstructed using the three‐dimensional reconstruction software NRecon (version 1.7.4.2, Bruker, Germany) and analyzed via a CT analyzer (version 1.18.8.0, Bruker). The bone mineral density (BMD), bone volume (BV), percentage bone volume (BV/TV), trabecular number (Tb.N), trabecular thickness (Tb.Th), and cortical thickness (Ct.Th), which are related to the bone mass and microstructure, were calculated and analyzed.

Micro‐CT‐based angiography was also performed. First, an intravenous needle was injected into the left ventricle for perfusion when the heart was exposed after anesthesia by intraperitoneal injection of 50 mg/kg of sodium pentobarbital (Sigma‐Aldrich). Then, 37°C normal saline supplemented with 10 IU/mL low‐molecular‐weight heparin was injected through the auricula dextra, followed by 4% paraformaldehyde and normal saline. Finally, the mice were housed overnight at 4°C after injection of Microfil solution at a rate of 3 mL/min. The next day, the right femurs were collected after the muscles and other excess soft tissues around the bone were removed and fixed again in 4% paraformaldehyde for 24 h. A 10% EDTA solution was used for decalcification before scanning using micro‐CT at 70 kV. NRecon (version 1.7.4.2, Bruker) and a CT Analyzer (version 1.18.8.0, Bruker) were used for reconstruction and analyses. The vascular volume fraction and blood vessel density were calculated to assess angiogenesis in the femoral heads.

### 2.8 Double calcein labeling

Double calcein labeling was conducted to detect the dynamic bone formation of the femoral heads. The calcein (10 mg/kg, Beyotime) that was dissolved in 2% sodium bicarbonate solution was administered intraperitoneally in the sixth week of modeling and injected again in an interval of 6 days. The femoral heads of the mice were removed and then fixed in 4% paraformaldehyde for 48 h. After fixation, the samples were dehydrated and embedded in methyl methacrylate (Macklin, Shanghai, China). The embedded samples were then cut into 10‐μm sections using a microtome (HistoCore AUTOCUT, Leica). Subsequently, images of the sections were obtained using a laser scanning confocal microscope (Olympus FV3000, Japan).

### 2.9 Statistical analysis

The results are presented as mean ± standard error of the mean. Statistical differences were analyzed using either Student's *t*‐test or one‐way analysis of variance (ANOVA) test. All statistical analyses were conducted using IBM SPSS Statistics 23 (IBM, New York, USA) and GraphPad Prism 9 (GraphPad, San Diego, USA). A significance level of *p* < 0.05 was considered statistically significant.

## 3 RESULTS

### 3.1 Decreased *ZEB1* expression and type‐H vessel formation were detected in the femoral heads of SONFH patients

**FIGURE 1:**
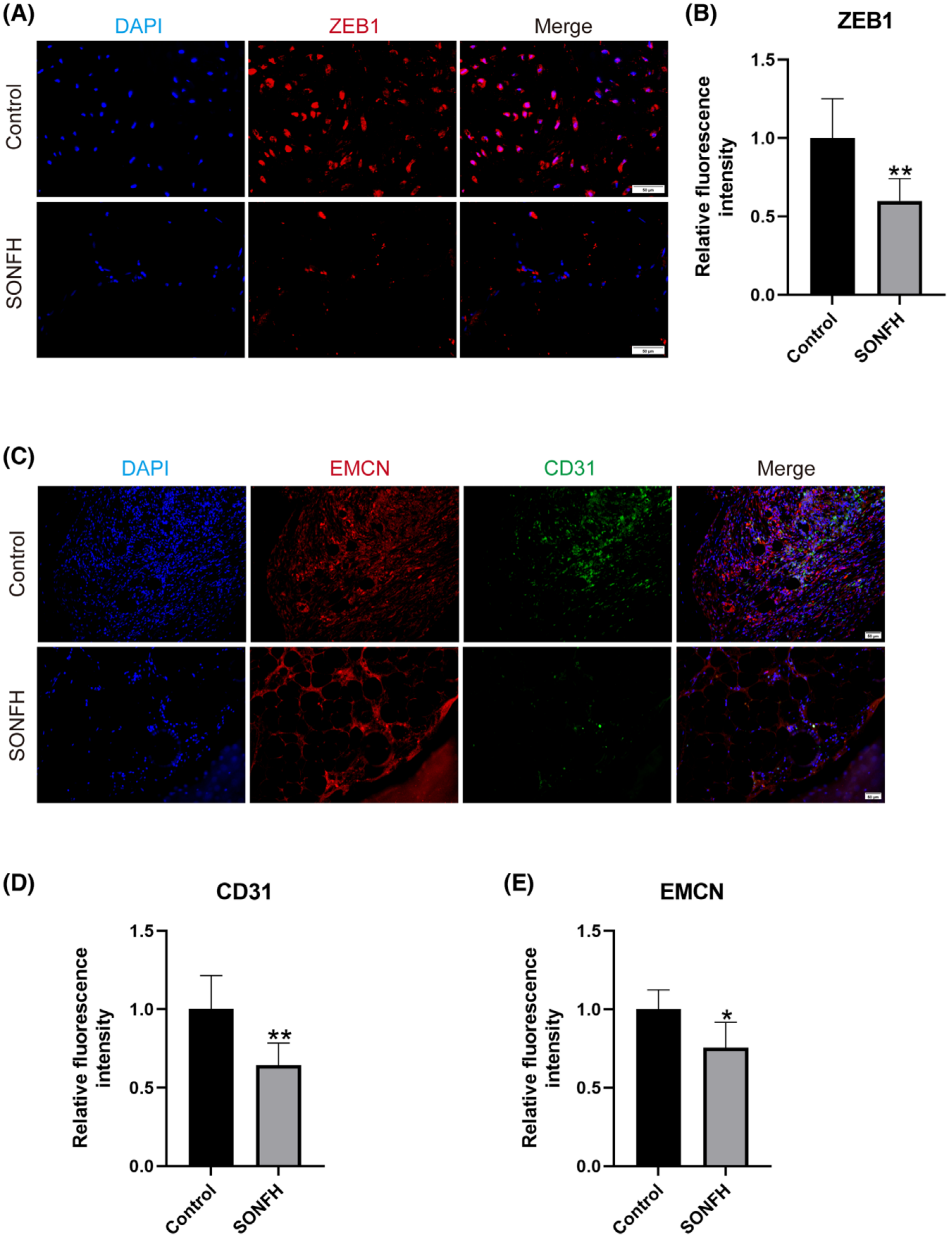
*ZEB1* and type‐H vessel expression are decreased in the femoral heads of SONFH patients. (A) Representative immunofluorescence (IF) images of *ZEB1* (red) in the femoral head. Scale bar: 50 μm. (B) Relative fluorescence intensity of *ZEB1*. (C) Representative IF images of *EMCN* (red) and *CD31* (green) in the femoral heads. Scale bar: 50 μm. (D, E) Relative fluorescence intensity of (D) *EMCN* and (E) *CD31*. *CD31*, platelet endothelial cell adhesion molecule‐1; *EMCN*, endomucin; SONFH, steroid‐induced osteonecrosis of the femoral head; *ZEB1*, zinc‐finger E‐box‐binding homeobox‐1. *p < 0.05, **p < 0.01. *Compared with the control group.

The femoral heads of SONFH and femur neck fracture patients were collected to detect the expression of *ZEB1* and the development of type‐H vessels. IF results demonstrated a significant decrease in the expression of *ZEB1* in the SONFH group compared to the control group (*p* < 0.01) (Figure 1A,B). Additionally, the expression levels of *EMCN* and *CD31*, which serve as biomarkers for type‐H vessels, were significantly lower in the SONFH group compared to the control group (*p* < 0.05) (Figure 1C–E). The findings indicate reduced expression levels of *ZEB1* and impaired formation of type‐H vessels in SONFH. Further investigations are required to elucidate the roles of these genes in the pathogenesis of SONFH.

### 3.2 SONFH and *ZEB1* knockdown models were successfully developed

**FIGURE 2:**
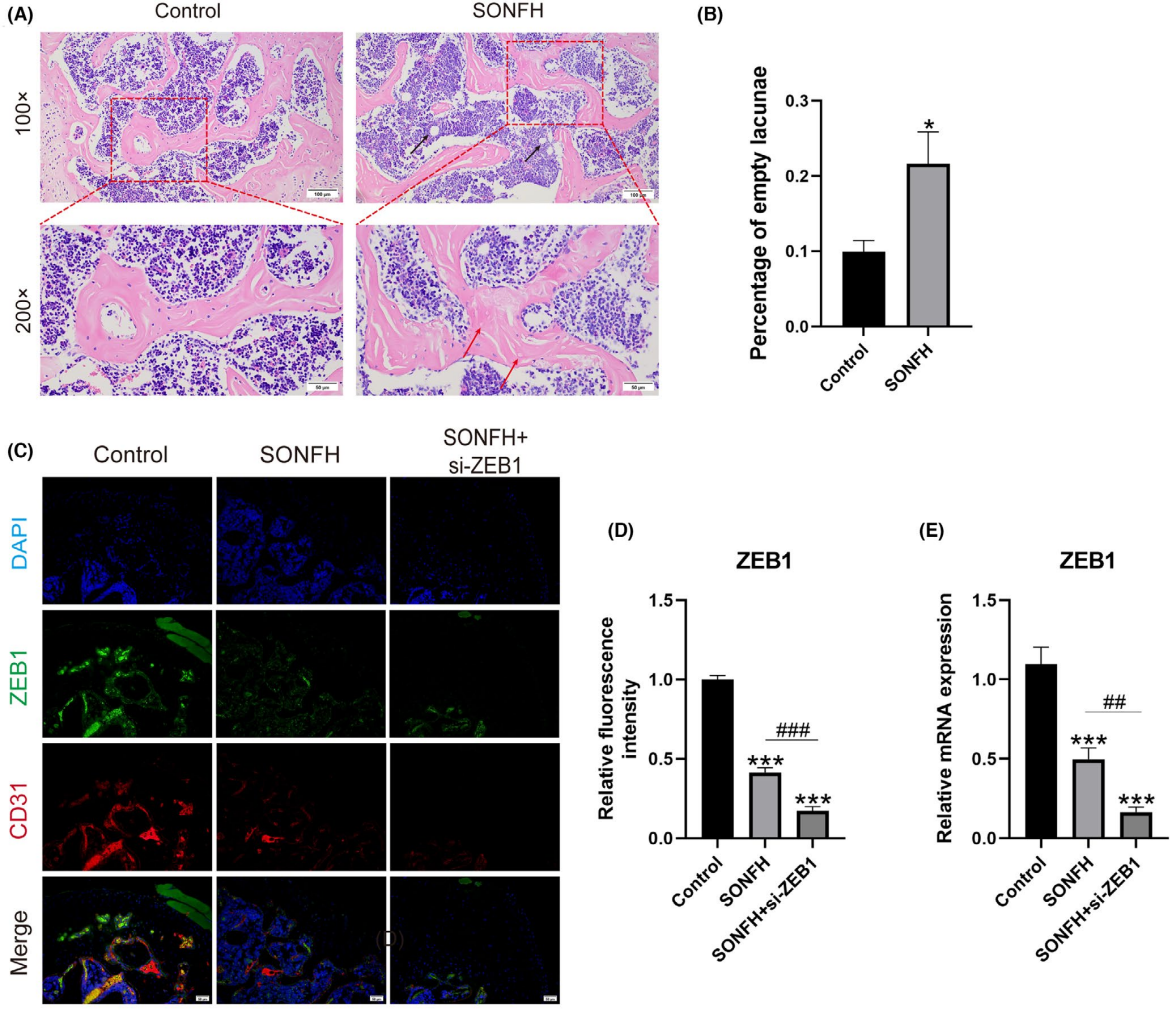
SONFH and *ZEB1* knockdown mouse models were successfully constructed. (A) The SONFH model was successfully constructed. Representative HE (hematoxylin and eosin)–stained images of mouse femoral heads are presented. The empty lacuna and lipoid degeneraria are indicated by red arrows and black arrows, respectively. Scale bar: 100 μm for 100× and 50 μm for 200×. (B) The percentage of empty lacunae was calculated. (C, D) *ZEB1* expression levels were measured using immunofluorescence. (E) The mRNA expression of *ZEB1* was detected using RT‐qPCR (quantitative real‐time polymerase chain reaction). *CD31*, platelet endothelial cell adhesion molecule‐1; SONFH, steroid‐induced osteonecrosis of the femoral head; *ZEB1*, zinc‐finger E‐box‐binding homeobox‐1. *p < 0.05 and ***p < 0.001; ^##^ *p* < 0.01 and ^###^ *p* < 0.001. *Compared with the control group, ^#^compared in the SONFH and SONFH+si‐*ZEB1* groups.

The SONFH model was successfully developed, and there was a decrease in the number of bone trabeculae in the femoral head. The amount of hematopoietic tissue in the bone marrow also decreased, and the tissue was replaced by adipocytes. Moreover, a significant increase in the proportion of empty lacunae was observed (Figure 2A,B). Then, the levels of *ZEB1* in the control, SONFH, and SONFH+si‐*ZEB1* groups were detected via IF and RT‐qPCR. A decreased expression of *ZEB1* in type‐H vessels was detected in the SONFH group and SONFH+si‐*ZEB1* group using IF (Figure 2C,D). Moreover, the total *ZEB1* level in the femoral heads of the mice was detected using RT‐qPCR. Low *ZEB1* levels were detected in the SONFH group (*p* < 0.001), and lower *ZEB1* levels were detected in the SONFH+si‐*ZEB1* group (*p* < 0.001) (Figure 2E), which indicated decreased *ZEB1* expression in SONFH patients and successful *ZEB1* knockdown in the SONFH+si‐ZEB1 group.

### 3.3 *ZEB1* could regulate the *Wnt/β‐catenin* pathway in vitro and in vivo

**FIGURE 3:**
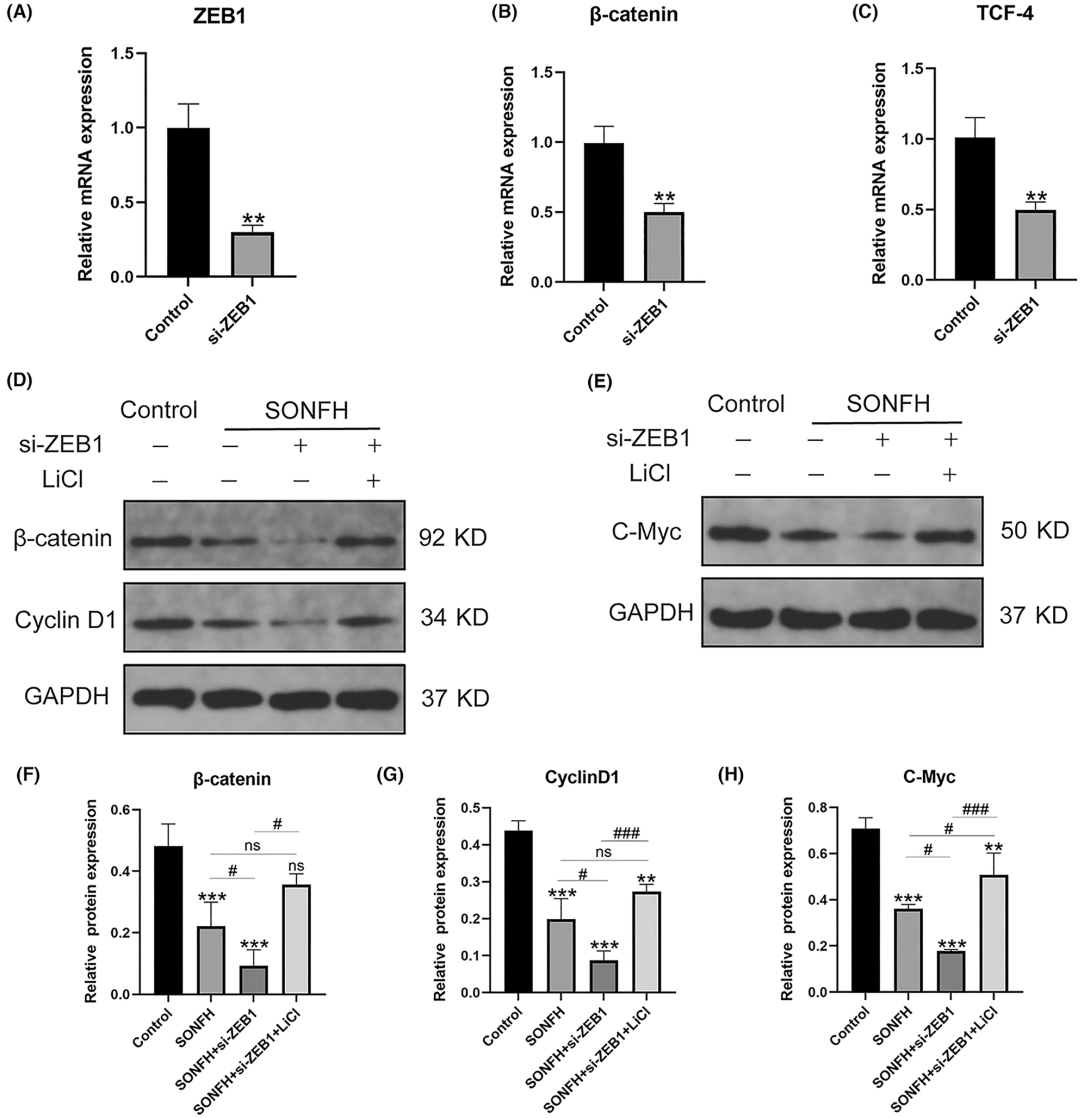
*ZEB1* could regulate the *Wnt/β‐catenin* pathway both in vitro and in vivo. (A) Relative mRNA (messenger RNA) expression of *ZEB1* in HUVECs (human umbilical vein endothelial cells). (B, C) The relative mRNA expression levels of *β‐catenin* and *TCF‐4* in HUVECs were measured. (D–H) Relative protein expression levels of *β‐catenin*, *cyclin D1*, and *C‐Myc* in SONFH mice. *TCF‐4*, transcription factor 4; SONFH, steroid‐induced osteonecrosis of the femoral head; *ZEB1*, zinc‐finger E‐box‐binding homeobox‐1. **p < 0.01, and ***p < 0.001; ^#^ *p* < 0.05 and ^###^ *p* < 0.001. *Compared with the control group, ^#^compared in the SONFH, SONFH+si‐*ZEB1*, and SONFH+si‐*ZEB1*+LiCl groups.

In vitro, HUVECs were successfully transfected with lentivirus to knock down *ZEB1*, which was confirmed using RT‐qPCR (Figure 3A). Then, the expression of *β‐catenin* and *TCF‐4*, key genes in the *Wnt/β‐catenin* pathway, was also detected using RT‐qPCR, and the results demonstrated that the expression of *β‐catenin* was significantly decreased after *ZEB1* was knocked down (*p* < 0.01), as shown in Figure 3B. Similarly, the expression level of *TCF‐4* was also decreased in the si‐*ZEB1* group (*p* < 0.01) (Figure 3C).

Moreover, to further examine the association between *ZEB1* and the *Wnt/β‐catenin* pathway in vivo, an activator of the *Wnt/β‐catenin* pathway was employed. As shown in Figure 3D–H, the expressions of *β‐catenin*, *cyclin D1*, and *C‐Myc* in the *Wnt/β‐catenin* pathway were lower in the SONFH group than in the control group and were greatly lower when the expression of *ZEB1* was decreased further (*p* < 0.001). Moreover, the downregulation of the *Wnt/β‐catenin* pathway caused by the decreased expression of *ZEB1* was reversed after the administration of LiCl (*p* < 0.001). Therefore, we concluded that a decrease in *ZEB1* could negatively regulate the *Wnt/β‐catenin* pathway.

### 3.4 Decreased *ZEB1* expression could reduce the formation of type‐H vessels via the *Wnt/β‐catenin* pathway

**FIGURE 4:**
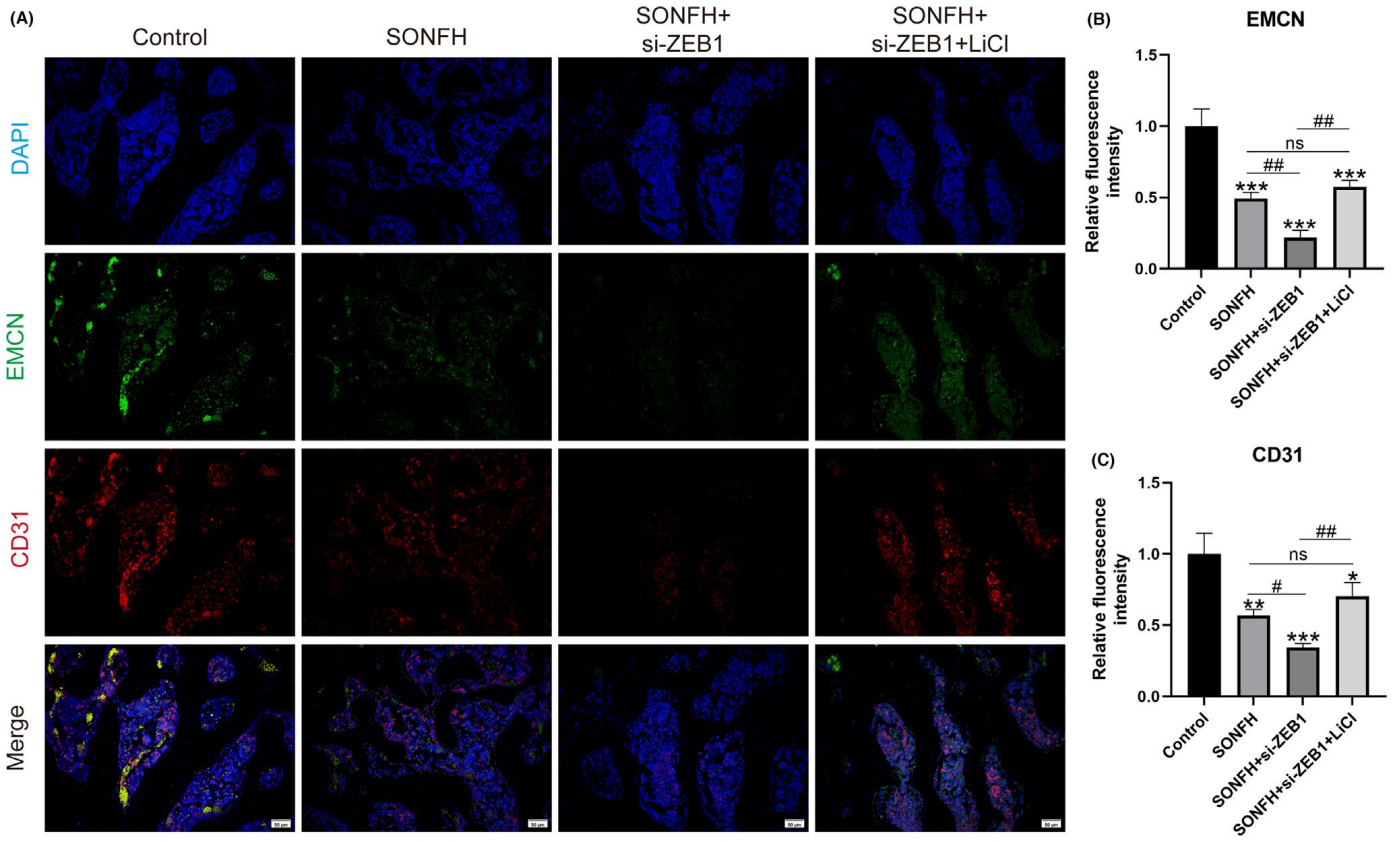
Decreased *ZEB1* expression could reduce the formation of type‐H vessels via the *Wnt/β‐catenin* pathway. (A) Representative immunofluorescence images showing a decrease in the number of type‐H vessels. Scale bar: 50 μm. (B) Quantitative calculation of the expression of *EMCN*. (C) Quantitative calculation of *CD31* expression. *CD31*, platelet endothelial cell adhesion molecule‐1; *EMCN*, endomucin; SONFH, steroid‐induced osteonecrosis of the femoral head; *ZEB1*, zinc‐finger E‐box‐binding homeobox‐1. *p < 0.05, **p < 0.01, and ***p < 0.001; ^#^ *p* < 0.05 and ^##^ *p* < 0.01. *Compared with the control group, ^#^compared in the SONFH, SONFH+si‐*ZEB1*, and SONFH+si‐*ZEB1*+LiCl groups.

It was found that *ZEB1* expression and type‐H vessel distribution decreased in SONFH patients, but the specific mechanism involved remains unclear. Therefore, *ZEB1* was knocked down to observe the development of type‐H vessels. The IF results showed that the expression of type‐H vessel‐related biomarkers was largely lower in the SONFH group than in the control group, and there were lower expression levels of type‐H vessel‐related biomarkers after *ZEB1* knockdown (Figure 4A–C). However, the expression levels of these biomarkers increased when the *Wnt/β‐catenin* pathway was activated (Figure 4A–C). Therefore, we concluded that the formation of type‐H vessels was mediated by the expression of *ZEB1* via the *Wnt/β‐catenin* pathway.

### 3.5 Decreased *ZEB1* expression could impair type‐H vessel‐mediated angiogenesis by regulating the *Wnt/β‐catenin* pathway

**FIGURE 5:**
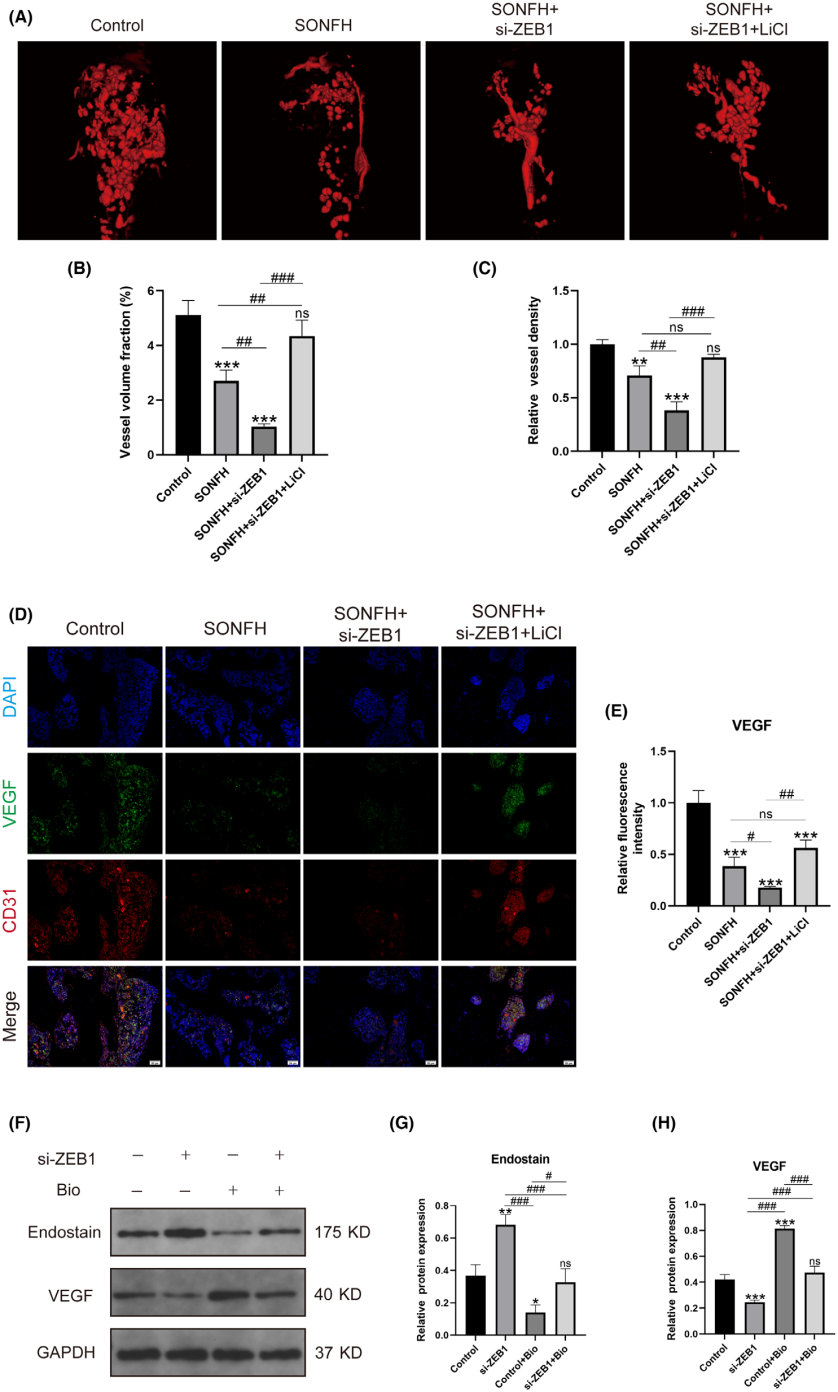
Type H‐mediated angiogenesis decreases due to a decrease in *ZEB1*, which causes impairment of the *Wnt/β‐catenin* pathway. (A) Representative micro‐CT (computed tomography)–based angiography images are presented. (B, C) The vessel volume fraction and relative vessel density were calculated. (D) Representative immunofluorescence images showing the expression of *VEGF* in type‐H vessels (yellow regions). Scale bar: 50 μm. (E) The levels of *VEGF* in all groups were calculated. (F–H) The expression levels of endostain and *VEGF* in HUVECs (human umbilical vein endothelial cells) were measured using Western blotting. *CD31*, platelet endothelial cell adhesion molecule‐1; SONFH, steroid‐induced osteonecrosis of the femoral head; *VEGF*, vascular endothelial growth factor; *ZEB1*, zinc‐finger E‐box‐binding homeobox‐1. *p < 0.05, **p < 0.01, and ***p < 0.001; ^#^ *p* < 0.05, ^##^ *p* < 0.01, and ^###^ *p* < 0.001. *Compared with the control group, ^#^compared in the SONFH, SONFH+si‐*ZEB1*, and SONFH+si‐*ZEB1*+LiCl groups; or in the si‐*ZEB1*,control+Bio and si‐*ZEB1*+Bio groups.

Micro‐CT‐based angiography was performed to further assess the number and density of vessels in the femoral heads. Figure 5A shows that there were fewer vessels in the SONFH group than in the control group, and the number of vessels further decreased after *ZEB1* was knocked down. However, there were more vessels in the SONFH+si‐*ZEB1* group when the *Wnt/β‐catenin* pathway was activated by LiCl (Figure 5A–C). Moreover, the level of *VEGF*, which can promote angiogenesis, was measured using IF. The findings indicated that the level of *VEGF* in the SONFH group was significantly lower than that in the control group (*p* < 0.001), and the decrease was even greater in the SONFH+si‐*ZEB1* group (*p* < 0.001). Conversely, the activation of the *Wnt/β‐catenin* pathway by LiCl led to a significant increase in *VEGF* expression (*p* < 0.001) (Figure 5D,E).

The expression of angiogenesis‐related genes, including endostatin (*ES*) and *VEGF*, was detected in HUVECs. *VEGF* promoted angiogenesis in HUVECs, whereas *ES* exhibited a negative effect. Western blotting results showed that, compared with those in the control group, the expression of *ES* was elevated, whereas the expression of *VEGF* was lower (Figure 5F–H), indicating that *ZEB1* knockdown impaired angiogenesis. However, treatment with BIO, a *Wnt/β‐catenin* pathway activator, significantly improved angiogenesis in HUVECs, as indicated by a decreased level of *ES* and increased expression of *VEGF* compared with those in the si‐*ZEB1* group (Figure 5F–H).

All these results suggested that the inhibition of *ZEB1* could attenuate angiogenesis. Conversely, activation of the *Wnt/β‐catenin* pathway could ameliorate impaired angiogenesis.

### 3.6 Decreased *ZEB1* expression could impair type‐H vessel‐mediated osteogenesis by regulating the Wnt/β‐catenin pathway

**FIGURE 6:**
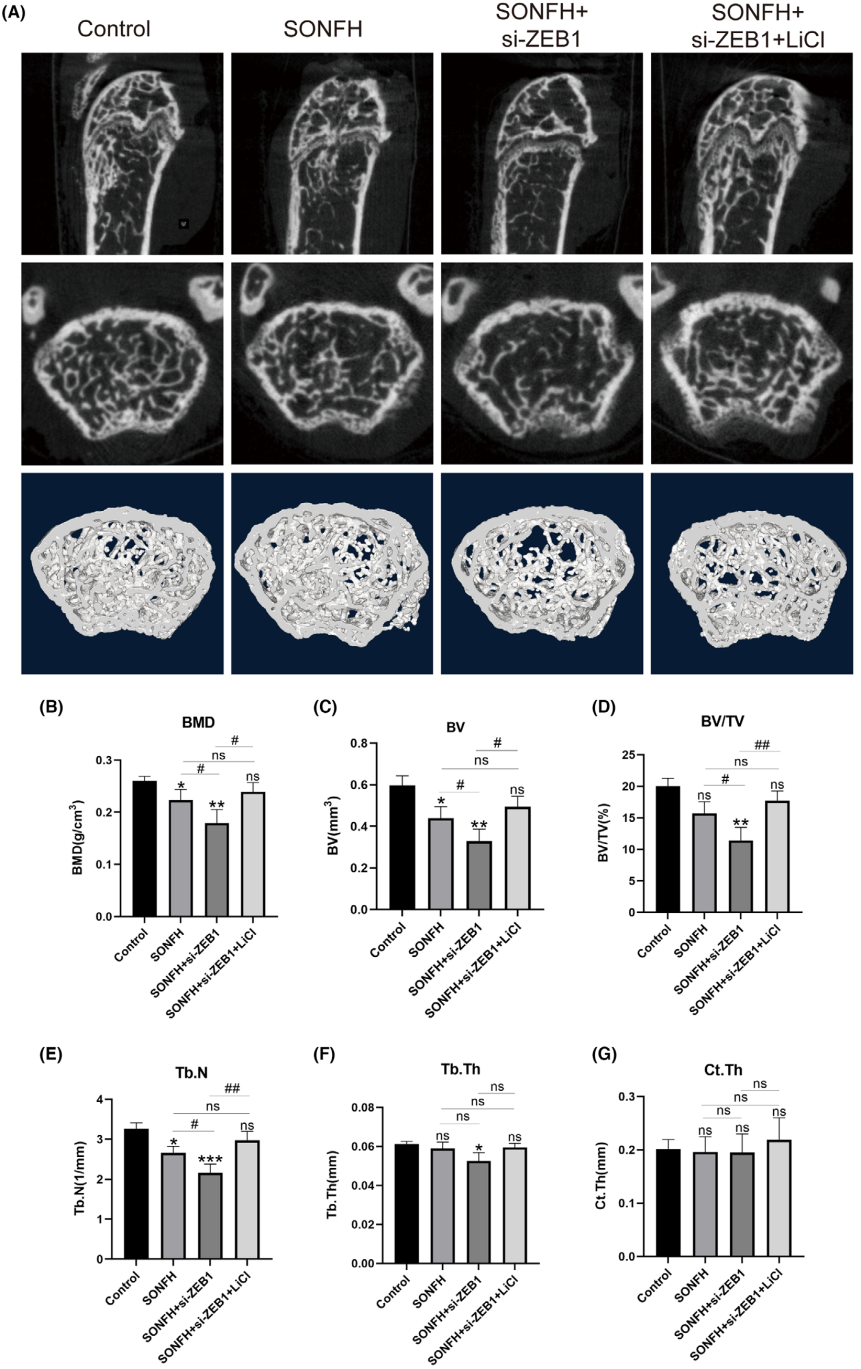
Micro‐CT (computed tomography) images and parameters for femoral heads in different groups. (A) Representative micro‐CT images of all groups. (B‐G) Micro‐CT parameters, including BMD, BV, BV/TV, Tb.N, Tb.Th, and Ct.Th, are presented. BMD, bone mineral density; BV, bone volume; BV/TV, percentage bone volume; Ct.Th, cortical thickness; SONFH, steroid‐induced osteonecrosis of the femoral head; Tb.N, trabecular number; Tb.Th, trabecular thickness; *ZEB1*, zinc‐finger E‐box‐binding homeobox‐1. *p < 0.05, **p < 0.01, and ***p < 0.001; ^#^ *p* < 0.05 and ^##^ *p* < 0.01. *Compared with the control group, ^#^compared in the SONFH, SONFH+si‐*ZEB1*, and SONFH+si‐*ZEB1*+LiCl groups.

The bone microstructure of the femoral heads was analyzed using micro‐CT and is shown in Figure 6A. Compared to the control group, the BMD, BV, BV/TV, and Tb.N in the SONFH group were significantly lower. These parameters were lower after *ZEB1* was knocked down (*p* < 0.05) but significantly increased when LiCl activated the *Wnt/β‐catenin* pathway (*p* < 0.05) (Figure 6B–E). These results showed that *ZEB1* could strongly reduce bone mass and change the microstructure of femoral heads. Furthermore, in the present study, we detected Tb.Th; however, no significant difference was observed in the SONFH group compared to the control group. In the si‐*ZEB1* group, the level of Tb.Th significantly decreased compared with that in the control group (*p* < 0.01) and improved substantially after the activation of the *Wnt/β‐catenin* pathway (*p* < 0.05) (Figure 6F), suggesting that only a substantial decrease in *ZEB1* might change the thickness of the trabecular bone. However, no significant change was found in any of the groups for Ct.Th (Figure 6G). These results revealed that the trabecular region was seriously affected by changes in *ZEB1* expression in the femoral heads.

**FIGURE 7:**
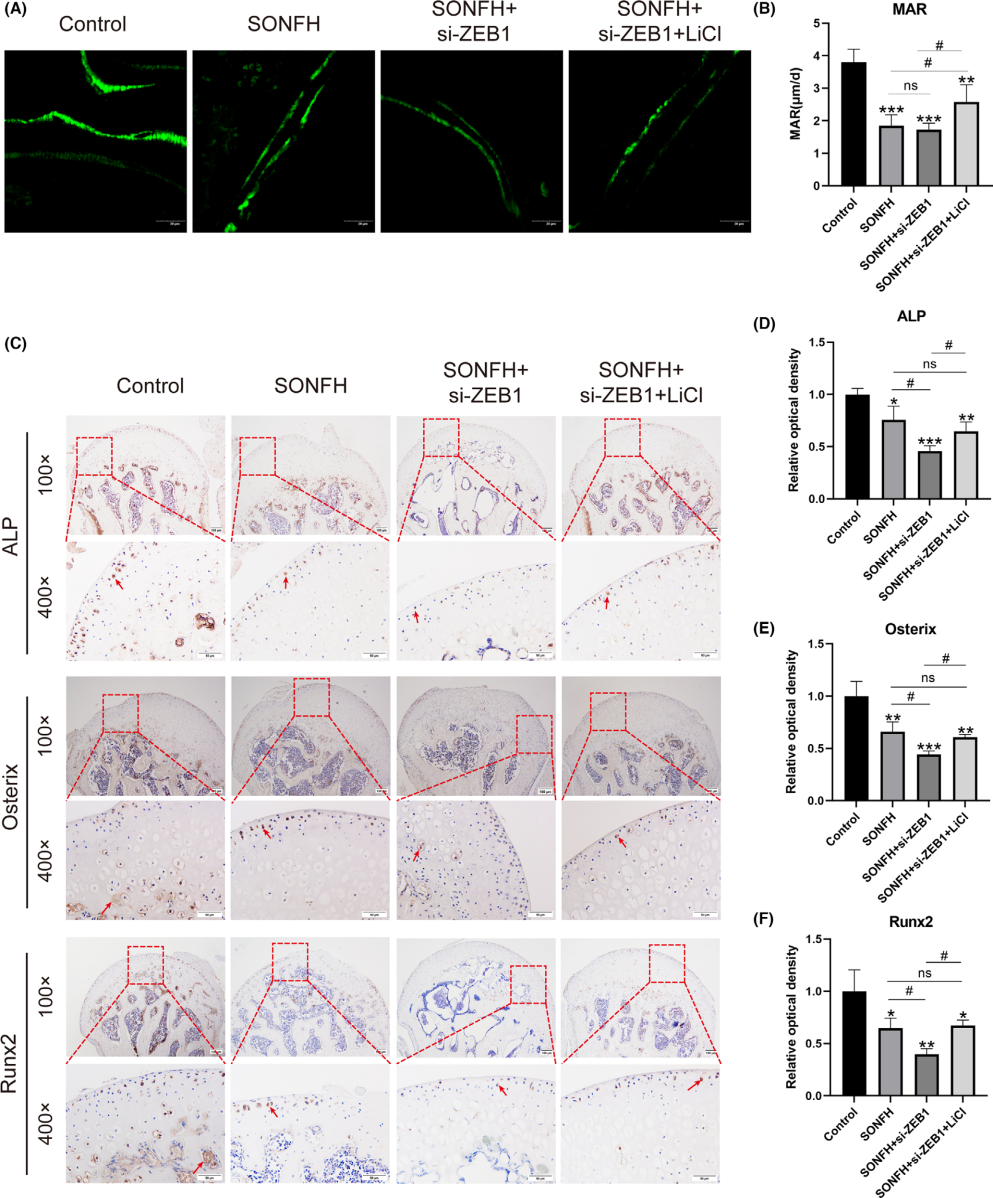
Type H‐mediated osteogenesis decreased due to the downregulation of *ZEB1*, which impaired the *Wnt/β‐catenin* pathway. (A) Representative image in all groups stained with calcein. The distance between two green labels was positively related to the bone mineralization rate. Scale bar: 20 μm. (B) MAR was calculated for all groups. (C–F) Representative immunohistochemical images and statistical analysis of *ALP*, *Osterix*, and *Runx2* expression. Scale bar: 100 μm for 100× and 50 μm for 400×. ALP, alkaline phosphatase; MAR, mineral apposition rate; *Runx2*, runt‐related transcription factor 2; SONFH, steroid‐induced osteonecrosis of the femoral head; *ZEB1*, zinc‐finger E‐box‐binding homeobox‐1. *p < 0.05, **p < 0.01, and ***p < 0.001; ^#^ *p* < 0.05 . *Compared with the control group, ^#^compared in the SONFH, SONFH+si‐*ZEB1*, and SONFH+si‐*ZEB1*+LiCl groups.

Additionally, the bone formation rate was assayed using double calcein labeling (Figure 7A). The results showed that the distance between the green signals in the SONFH and the SONFH+si‐*ZEB1* groups was shorter than that in the control and the SONFH+si‐*ZEB1*+LiCl groups. The mineral apposition rate (MAR), defined as the ratio of distance to time, was calculated to quantify the bone formation rate. Similarly, compared with that in the SONFH group, the MAR in the SONFH and the SONFH+si‐*ZEB1* groups significantly decreased (*p* < 0.001), but no significant difference was detected when *ZEB1* was knocked down. After the *Wnt/β‐catenin* pathway was activated, the MAR significantly increased compared with that in the SONFH+si‐*ZEB1* group (*p* < 0.05) (Figure 7B). The expression of osteogenesis‐related genes, including *ALP*, *Osterix*, and *Runx2*, was also measured using IHC (Figure 7C–F). ALP expression was significantly decreased in the SONFH group compared to the control group, and this reduction was further enhanced after the knockdown of *ZEB1* (*p* < 0.01). However, the application of LiCl resulted in a significant increase in *ALP* expression. Additionally, the expression of *Osterix* and *Runx2* exhibited similar patterns.

## 4 DISCUSSION

Currently, prolonged and widespread use of glucocorticoids has emerged as a key risk factor for the development of ONFH. Although core decompression, vascularized bone grafts, and other methods have been applied for the treatment of SONFH, the therapeutic efficacy of these methods has been unsatisfactory, and the major procedure for treating SONFH is total hip arthroplasty during the late period, which places great burdens on individuals and society.^20^ Therefore, it is essential to explore the mechanism of this disease and put in place early intervention measures. In this study, we confirmed that a reduction in *ZEB1* expression can hinder the *Wnt/β‐catenin* pathway, causing a decrease in the development of type‐H vessels. This impairment of angiogenesis and osteogenesis coupling facilitated by type‐H vessels ultimately speeds up the progression of SONFH. These findings provide a fresh perspective on the pathogenesis of SONFH.

*ZEB1* is strongly associated with the *Wnt/β‐catenin* pathway in numerous diseases. It was demonstrated that *ZEB1* could trigger the activation of the *Wnt/β‐catenin* pathway by repressing *Wif1*, ultimately alleviating vascular deformities.^21^ Moreover, Zhao et al. demonstrated that *ZEB1* could enhance the transcription of *ELK3*, a member of the ternary complex factor, to stimulate the activation of the *Wnt/β‐catenin* pathway.^22^ Another study revealed that *ZEB1* could regulate the *Wnt/β‐catenin* pathway to act on insubstantial hepatic stellate cells and liver fibrosis.^23^ In the present study, the impaired *Wnt/β‐catenin* pathway was identified in SONFH, and this pathway was further inhibited after *ZEB1* was knocked down. However, the specific underlying mechanism is unclear and requires additional research.

Type‐H vessels, distinguished by elevated expression levels of *EMCN* and *CD31*, are predominantly situated in close proximity to the growth plate at the metaphyseal end and within the periosteum of bone. These vessels are virtually absent in other tissues and play a crucial role in facilitating the coordination of angiogenesis and osteogenesis.^4^ *ZEB1* was predominantly detected in type‐H vessels, and the lack of *ZEB1* in endothelial cells could decrease the development of type‐H vessels and bone.^15^ Additionally, stimulating *ZEB1/Notch1* facilitates the development of type‐H vessels and orchestrates the integration of osteogenesis and angiogenesis, ultimately promoting bone regeneration.^17^ In the present study, decreased levels of *ZEB1* were detected in the femoral heads of SONFH patients and mice. Moreover, the quantity of type‐H vessels and the degree of coupling between angiogenesis and osteogenesis decreased after the knockdown of *ZEB1* in the femoral heads of mice. Although there are a few studies on the role of *ZEB1* in SONFH, the functions of *ZEB1* in the formation of bones and vessels have been well illustrated. For example, *ZEB1* was found to promote the generation of osteoblasts and inhibit the differentiation of osteoclasts, ultimately reducing the occurrence of postmenopausal osteoporosis.^24^ Similarly, Tao et al. constructed a polymeric coating on a β‐tricalcium phosphate scaffold loaded with *ZEB1* and demonstrated that *ZEB1* could stimulate bone defect healing by enhancing angiogenesis and osteogenesis while suppressing osteoclast formation.^25^ However, *ZEB1* was also found to block osteoblastic differentiation during normal bone development.^26^ Other researchers found that silencing *ZEB1* in BMSCs enhanced osteogenic function in vitro, whereas there was an inverse relationship between *ZEB1* expression and bone mass as well as bone formation in postmenopausal women in vivo.^27^ The differences in the conclusions regarding the roles of *ZEB1* among various studies might be attributed to differences in the subjects and animal models used. The present study revealed that a decrease in *ZEB1* might accelerate the process of SONFH by inhibiting angiogenesis and osteogenesis.

In this study, we detected the formation of type‐H vessels in the femoral heads and found decreased expression in SONFH patients and in a mouse model. Type‐H vessels have been indicated to reduce bone loss and promote bone generation in previous studies.^28^, ^29^ Although platelet‐derived growth factor‐BB was found to promote reparative osteogenesis in SONFH by targeting type‐H vessels,^30^ studies exploring the roles of type‐H vessels in SONFH are rare. Furthermore, our research revealed that the coordination between angiogenesis and osteogenesis was impaired in patients with SONFH, attributed to the reduced quantity of type‐H vessels. Our initial investigation demonstrated that *ES* could suppress angiogenesis and osteogenesis in the femoral head, expediting the progression of SONFH.^31^ Li et al. constructed a scaffold and revealed that it could ameliorate SONFH by enhancing angiogenesis and bone regeneration via the *Wnt/β‐catenin* pathway.^32^ In another previous study, after combination of the parathyroid hormone and core compression, there was an increase in the number of vessels within the tunnels, along with elevated expression levels of *BMP‐2*, *Runx2*, and *VEGF* in SONFH rabbits.^33^ Moreover, Sun et al. revealed a decrease in the MAR, BMD, BV/TV, and Tb.N in SONFH, as detected using double calcein labeling and micro‐CT, which was consistent with our research.^34^ Nevertheless, these studies have mainly concentrated on impaired bone or vessel formation rather than the coordination of vascularization and bone formation, which are regulated by type‐H vessels.

This current study has several limitations. First, a decrease in *ZEB1* was shown to hinder the *Wnt/β‐catenin* pathway and diminish the formation of type‐H vessels, resulting in the development of SONFH. Conversely, administering an activator of the *Wnt/β‐catenin* pathway could alleviate these conditions. We subsequently attempted to upregulate the expression of *ZEB1*, but the constructed model was unstable and could not be used for further analysis. Therefore, it remains unclear whether the upregulation of *ZEB1* expression can delay the development of SONFH due to our inability to establish a model for *ZEB1* overexpression. Additionally, we proved that *ZEB1* could affect type‐H vessels via the Wnt/β‐catenin pathway, but the specific mechanism through which *ZEB1* acts on the *Wnt/β‐catenin* pathway is unclear and requires additional research.

This study revealed that the expression of *ZEB1* and the formation of type‐H vessels in the femoral heads decreased in SONFH. Further analysis revealed that a decrease in *ZEB1* could impair the formation of type‐H vessels and mediate angiogenesis and osteogenesis via the *Wnt/β‐catenin* pathway. Additionally, the application of LiCl activated the *Wnt/β‐catenin* pathway and enhanced the number of type‐H vessels, ultimately alleviating SONFH, which provides a novel strategy for clarifying the pathogenesis of SONFH.

## AUTHOR CONTRIBUTIONS

**Guangyang Zhang:** Conceptualization; formal analysis; methodology; writing – original draft. **Yuanqing Cai:** Data curation; writing – original draft. **Jialin Liang:** Resources; software. **Zhaopu Jing:** Investigation; resources. **Wang Wei:** Data curation; visualization. **Leifeng Lv:** Software; validation; visualization. **Xiaoqian Dang:** Funding acquisition; methodology; writing – review and editing. **Qichun Song:** Formal analysis; methodology; writing – review and editing.

## FUNDING INFORMATION

This study received funding from the National Natural Science Foundation of China (grant number 82002311), the China Postdoctoral Science Foundation (grant number 2021M692575), and the Fundamental Research Funds for the Central Universities (grant number xzy022024016).

## CONFLICT OF INTEREST STATEMENT

The authors confirm that the research was conducted without any commercial or financial relationships that could be perceived as a potential conflict of interest.

## ETHICS APPROVAL AND CONSENT TO PARTICIPATE

The animal study was reviewed and approved by the Ethics Committee of Xi'an Jiao Tong University Health Science Center (2020–938), and the clinical research was approved by the Ethics Committee of the Second Affiliated Hospital of Xi'an Jiaotong University (2023213). The investigation was carried out following the rules of the Declaration of Helsinki.

## PATIENT CONSENT FOR PUBLICATION

All patients participating in this research provided consent for publication, and written informed consent was obtained from all individuals. All procedures conducted in this study adhered to the applicable guidelines and regulations.

## Supporting information


Table S1.

## References

[1] Mont MA , Cherian JJ , Sierra RJ , Jones LC , Lieberman JR . Nontraumatic osteonecrosis of the femoral head: where do we stand today? A ten‐year update. J Bone Joint Surg Am. 2015;97:1604‐1627. 26446969 10.2106/JBJS.O.00071

[2] Zhao D‐W , Yu M , Hu K , et al. Prevalence of nontraumatic osteonecrosis of the femoral head and its associated risk factors in the Chinese population: results from a nationally representative survey. Chin Med J. 2015;128:2843‐2850. 26521779 10.4103/0366-6999.168017 PMC4756878

[3] Wang A , Ren M , Wang J . The pathogenesis of steroid‐induced osteonecrosis of the femoral head: a systematic review of the literature. Gene. 2018;671:103‐109. 29859289 10.1016/j.gene.2018.05.091

[4] Peng Y , Wu S , Li Y , Crane JL . Type‐H blood vessels in bone modeling and remodeling. Theranostics. 2020;10:426‐436. 31903130 10.7150/thno.34126 PMC6929606

[5] Chen Y , Yin Y , Luo M , et al. Occlusal force maintains alveolar bone homeostasis via type‐H angiogenesis. J Dent Res. 2023;102(12): 1356‐1365. 37786932 10.1177/00220345231191745

[6] Li J , Wu G , Xu C , et al. SLIT guidance ligand 3 (SLIT3) loaded in hydrogel microparticles enhances the tendon‐bone healing through promotion of type‐H vessel formation: an experimental study in mice. Int J Mol Sci. 2;13638. 10.3390/ijms241713638 PMC10488208 37686444

[7] Ruan Z , Yin H , Wan T‐F , et al. Metformin accelerates bone fracture healing by promoting type‐H vessel formation through inhibition of YAP1/TAZ expression. Bone Res. 2023;11:45. 37587136 10.1038/s41413-023-00279-4 PMC10432554

[8] Qiu M , Li C , Cai Z , et al. 3D biomimetic calcified cartilaginous callus that induces type‐H vessels formation and Osteoclastogenesis. Adv Sci (Weinh). 2023;10:e2207089. 36999832 10.1002/advs.202207089 PMC10238192

[9] Huang L , Wang Y , Jiang Y , Wu Y , Hu C , Ouyang H . High levels of GSK‐3β signalling reduce osteogenic differentiation of stem cells in osteonecrosis of femoral head. J Biochem. 2018;163:243‐251. 29136173 10.1093/jb/mvx076

[10] Xu H , Fang L , Zeng Q , et al. Glycyrrhizic acid alters the hyperoxidative stress‐induced differentiation commitment of MSCs by activating the Wnt/β‐catenin pathway to prevent SONFH. Food Funct. 2023;14:946‐960. 36541285 10.1039/d2fo02337g

[11] Zhang S , Dong K , Zeng X , Wang F , Lu M . Astragalus polysaccharide ameliorates steroid‐induced osteonecrosis of the femoral head by regulating miR‐200b‐3p‐mediated Wnt/β‐catenin signaling pathway via inhibiting SP1 expression: astragalus polysaccharide regulates SONFH via SP1. BMC Musculoskelet Disord. 2023;24:369. 37165386 10.1186/s12891-023-06447-1 PMC10170750

[12] Shen J , Sun Y , Liu X , et al. EGFL6 regulates angiogenesis and osteogenesis in distraction osteogenesis via Wnt/β‐catenin signaling. Stem Cell Res Ther. 2021;12:415. 34294121 10.1186/s13287-021-02487-3 PMC8296592

[13] Wang X , Ma Y , Chen J , et al. A novel decellularized matrix of Wnt signaling‐activated osteocytes accelerates the repair of critical‐sized parietal bone defects with osteoclastogenesis, angiogenesis, and neurogenesis. Bioact Mater. 2023;21:110‐128. 36093329 10.1016/j.bioactmat.2022.07.017 PMC9411072

[14] Fu R , Han C‐F , Ni T , et al. A ZEB1/p53 signaling axis in stromal fibroblasts promotes mammary epithelial tumours. Nat Commun. 2019;10:3210. 31324807 10.1038/s41467-019-11278-7 PMC6642263

[15] Fu R , Lv W‐C , Xu Y , et al. Endothelial ZEB1 promotes angiogenesis‐dependent bone formation and reverses osteoporosis. Nat Commun. 2020;11:460. 31974363 10.1038/s41467-019-14076-3 PMC6978338

[16] Zeng K , Xie W , Wang C , et al. USP22 upregulates ZEB1‐mediated VEGFA transcription in hepatocellular carcinoma. Cell Death Dis. 2023;14:194. 36906615 10.1038/s41419-023-05699-y PMC10008583

[17] Zhou J , Li Y , He J , et al. ROS scavenging graphene‐based hydrogel enhances type‐H vessel formation and vascularized bone regeneration via ZEB1/Notch1 mediation. Macromol Biosci. 2023;23:e2200502. 36637816 10.1002/mabi.202200502

[18] Sánchez‐Tilló E , de Barrios O , Valls E , Darling DS , Castells A , Postigo A . ZEB1 and TCF4 reciprocally modulate their transcriptional activities to regulate Wnt target gene expression. Oncogene. 2015;34:5760‐5770. 26387539 10.1038/onc.2015.352

[19] Li L‐Y , Yang J‐F , Rong F , et al. ZEB1 serves an oncogenic role in the tumourigenesis of HCC by promoting cell proliferation, migration, and inhibiting apoptosis via Wnt/β‐catenin signaling pathway. Acta Pharmacol Sin. 2021;42:1676‐1689. 33514855 10.1038/s41401-020-00575-3 PMC8463676

[20] Wang J , Xu P , Zhou L . Comparison of current treatment strategy for osteonecrosis of the femoral head from the perspective of cell therapy. Front Cell Dev Biol. 2023;11:995816. 37035246 10.3389/fcell.2023.995816 PMC10073660

[21] Yu QC , Geng A , Preusch CB , et al. Activation of Wnt/β‐catenin signaling by Zeb1 in endothelial progenitors induces vascular quiescence entry. Cell Rep. 2022;41:111694. 36417861 10.1016/j.celrep.2022.111694

[22] Zhao Q , Ren Y , Xie H , et al. ELK3 mediated by ZEB1 facilitates the growth and metastasis of pancreatic carcinoma by activating the Wnt/β‐catenin pathway. Front Cell Dev Biol. 2021;9:700192. 34409034 10.3389/fcell.2021.700192 PMC8365240

[23] Yang J , Tao Q , Zhou Y , et al. MicroRNA‐708 represses hepatic stellate cells activation and proliferation by targeting ZEB1 through Wnt/β‐catenin pathway. Eur J Pharmacol. 2020;871:172927. 31962101 10.1016/j.ejphar.2020.172927

[24] Zhu X , Yan F , Liu L , Huang Q . ZEB1 regulates bone metabolism in osteoporotic rats through inducing POLDIP2 transcription. J Orthop Surg Res. 2022;17:423. 36123704 10.1186/s13018-022-03312-0 PMC9484217

[25] Tao S‐C , Li X‐R , Wei W‐J , et al. Polymeric coating on β‐TCP scaffolds provides immobilization of small extracellular vesicles with surface‐functionalization and ZEB1‐loading for bone defect repair in diabetes mellitus. Biomaterials. 2022;283:121465. 35286850 10.1016/j.biomaterials.2022.121465

[26] Ruh M , Stemmler MP , Frisch I , et al. The EMT transcription factor ZEB1 blocks osteoblastic differentiation in bone development and osteosarcoma. J Pathol. 2021;254:199‐211. 33675037 10.1002/path.5659

[27] Xu C , Shi H , Jiang X , et al. ZEB1 mediates bone marrow mesenchymal stem cell osteogenic differentiation partly via Wnt/β‐catenin signaling. Front Mol Biosci. 2021;8:682728. 34109218 10.3389/fmolb.2021.682728 PMC8183571

[28] Xu Z , Kusumbe AP , Cai H , Wan Q , Chen J . Type‐H blood vessels in coupling angiogenesis‐osteogenesis and its application in bone tissue engineering. J Biomed Mater Res B Appl Biomater. 2023;111:1434‐1446. 36880538 10.1002/jbm.b.35243

[29] Li J , Wei G , Liu G , et al. Regulating type‐H vessel formation and bone metabolism via bone‐targeting Oral micro/Nano‐hydrogel microspheres to prevent bone loss. Adv Sci (Weinh). 2023;10:e2207381. 36967561 10.1002/advs.202207381 PMC10214236

[30] Cao H , Shi K , Long J , et al. PDGF‐BB prevents destructive repair and promotes reparative osteogenesis of steroid‐associated osteonecrosis of the femoral head in rabbits. Bone. 2023;167:116645. 36539110 10.1016/j.bone.2022.116645

[31] Zhao Y , Li D , Duan D‐P , Song Q‐C . The effect of endostatin on angiogenesis and osteogenesis of steroid‐induced osteonecrosis of the femoral head in a rabbit model. Acta Orthop Traumatol Turc. 2022;56:178‐186. 35703505 10.5152/j.aott.2022.21248 PMC9612648

[32] Li B , Lei Y , Hu Q , Li D , Zhao H , Kang P . Porous copper‐ and lithium‐doped nano‐hydroxyapatite composite scaffold promotes angiogenesis and bone regeneration in the repair of glucocorticoids‐induced osteonecrosis of the femoral head. Biomed Mater. 2021;16:065012. 10.1088/1748-605X/ac246e 34492640

[33] Zhou C‐H , Meng J‐H , Zhao C‐C , et al. PTH[1‐34] improves the effects of core decompression in early‐stage steroid‐associated osteonecrosis model by enhancing bone repair and revascularization. PLoS One. 2017;12:e0178781. 28562696 10.1371/journal.pone.0178781 PMC5451136

[34] Sun H , Zhang W , Yang N , et al. Activation of cannabinoid receptor 2 alleviates glucocorticoid‐induced osteonecrosis of femoral head with osteogenesis and maintenance of blood supply. Cell Death Dis. 2021;12:1035. 34718335 10.1038/s41419-021-04313-3 PMC8556843

