# Supplementary material for: The decrease in zinc‐finger E‐box‐binding homeobox‐1 could accelerate steroid‐induced osteonecrosis of the femoral head by repressing type‐H vessel formation via Wnt/β‐catenin pathway
Source: Animal Model Exp Med. 2024 Dec 16;7(6):802–15. doi: 10.1002/ame2.12507 (PMC11680474; doi:10.1002/ame2.12507)
Supplement: Supplementary file 1 — Table S1. [file AME2-7-802-s001.pdf]

Table S1 Primers for RT-qPCR

Gene	Forward Primer	Reverse Primer
Homo GAPDH	TCAAGAAGGTGGTGAAGCAGG	TCAAAGGTGGAGGAGTGGGT
Homo β-catenin	GAAACGGCTTTCAGTTGAGC	CTGGCCATATCCACCAGAGT
Homo ZEB1	TATGAATGCCCAAACTGCAA	TGGTGATGCTGAAAGAGACG
Homo TCF	TCCATCAGCAAGCACTGCCGACTA	TTGCCCAACATTCCTGCATAGCC
Mus GAPDH	ATGGGTGTGAACCACGAGA	CAGGGATGATGTTCTGGGCA
Mus ZEB1	TCAGTGTTCCTCGCCATCTCTT	CCACCACTGCTAAAAACCCCAT

GAPDH, Glyceraldehyde 3-phosphate dehydrogenase; ZEB1, Zinc-finger E-box-binding homeobox-1; TCF, Transcription factor 4.

Table S2 Antibodies for WB, IF and IHC staining

Experiment	Antibody Name	Supplier	Code
WB	GAPDH	Abcam	Ab8245
	VEGF	Abcam	Ab214424
	Endostatin	LSBio	LS-C806230
	β-catenin	Affinity	AF6266
	Cyclin D1	Proteintech	60186-1-Ig
	C-Myc	Proteintech	10828-1-AP
IF	EMCN (Human)	Affinity	DF13357
	EMCN (Mouse)	Abcam	ab106100
	CD31(Human)	abcam	ab9498
	CD31 (Mouse)	Proteintech	66065-2-Ig
	ZEB1	CST	70512S
IHC	VEGF	Affinty	AF5131
	ALP	Proteintech	11187-1-AP
	Osterix	Abcam	ab209484
	Runx2	Abcam	Ab192256

GAPDH, Glyceraldehyde 3-phosphate dehydrogenase; VEGF, vascular endothelial growth factor; EMCN, Endomucin; CD31, platelet endothelial cell adhesion molecule-1; ZEB1, Zinc-finger E-box-binding homeobox-1; ALP, alkaline phosphatase; Runx2, runt-related transcription factor 2.
